# Metastases of primary mixed no-special type and lobular breast cancer display an exclusive lobular histology

**DOI:** 10.1016/j.breast.2024.103732

**Published:** 2024-04-12

**Authors:** Gitte Zels, Karen Van Baelen, Maxim De Schepper, Kristien Borremans, Tatjana Geukens, Edoardo Isnaldi, Hava Izci, Sophia Leduc, Amena Mahdami, Marion Maetens, Ha Linh Nguyen, Anirudh Pabba, François Richard, Josephine Van Cauwenberge, Ann Smeets, Ines Nevelsteen, Patrick Neven, Hans Wildiers, Wouter Van Den Bogaert, Giuseppe Floris, Christine Desmedt

**Affiliations:** a Laboratory for Translational Breast Cancer Research, Department of Oncology, KU Leuven, Belgium; b Department of Pathology, UZ Leuven, Belgium; c Gynecological Oncology Unit, Department of Gynecology, University Hospitals Leuven, Belgium; d General Medical Oncology Unit, Department of Oncology, University Hospitals Leuven, Belgium; e Department of Surgical Oncology, University Hospitals Leuven, Leuven, Belgium; f Department of Forensic Medicine, University Hospitals Leuven, Leuven, Belgium; g Laboratory for Translational Cell & Tissue Research, Department of Imaging & Pathology, KU Leuven, Belgium

**Keywords:** mixed invasive breast cancer of no-special type and invasive lobular cancer, Metastatic disease, E-cadherin

## Abstract

Primary tumors with a mixed invasive breast carcinoma of no-special type (IBC-NST) and invasive lobular cancer (ILC) histology are present in approximately five percent of all patients with breast cancer and are understudied at the metastatic level. Here, we characterized the histology of metastases from two patients with primary mixed IBC-NST/ILC from the postmortem tissue donation program UPTIDER (NCT04531696). The 14 and 43 metastatic lesions collected at autopsy had morphological features and E-cadherin staining patterns consistent with pure ILC. While our findings still require further validation, they may challenge current clinical practice and imaging modalities used in these patients.

## Highlights

• Approximately 5 percent of primary breast cancer consist of a mixture of NST and ILC.
• Mixed IBC-NST/ILC is understudied, especially in the metastatic setting.
• We collected metastases of 2 patients with primary mixed IBC-NST/ILC via rapid autopsies.
• All metastases of these patients displayed a lobular histology.
• Awareness of symptoms related to ILC is needed in patients with mixed IBC-NST/ILC.

## 1 Introduction

The majority of breast cancer (BC) cases are diagnosed as invasive breast carcinoma of no-special type (IBC-NST), previously known as invasive ductal carcinoma, whereas approximately 15 % of cases are diagnosed as invasive lobular carcinoma (ILC) [1]. It is known that ILC is associated with many differences on a clinical and biological level as compared to IBC-NST, with the hallmark being a discohesive histological growth pattern due to a lack or deficiency of E-cadherin [2]. Both types metastasize to bone, brain, lung, and liver. However, ILC additionally spreads more often to the gastro-intestinal tract, genitourinary tract, leptomeninges and peritoneum [3]. This peculiar metastatic spread remains often undetected by standard imaging techniques, like computed tomography (CT) and [18F]2-fluoro-2-deoxy-d-glucose positron emission tomography (18F-FDG PET)/CT [4]. Other techniques, like whole-body diffusion weighted magnetic resonance imaging (WB-DWI MRI), have been proposed in patients with ILC to improve the detection of metastases [[5], [6], [7]].

In most patients, IBC-NST and ILC are found in a pure form in the primary tumor. However, approximately five percent of all primary BC consist of a mixture of IBC-NST and ILC [8,9]. Throughout the years the definition of mixed IBC-NST/ILC changed and according to the latest WHO classification of breast tumors, 5th edition 2019, this type is defined by the representation of at least 10 % of the tumor by both components [[10], [11], [12]]. The diagnosis of mixed IBC-NST/ILC is typically made by combining histopathological examination on hematoxylin-eosin stain (H&E) with E-cadherin IHC, aiding in visualizing both components [12].

As compared to IBC-NST, mixed IBC-NST/ILC tumors are associated with lower grade, more lymph node positivity, larger tumor size, higher likelihood of hormone sensitivity and lack of *HER2* amplification [10]. In comparison to ILC, mixed IBC-NST/ILC is found in younger patients and the tumors have a higher histological grade and are less likely to be estrogen receptor-positive (ER+). Although clinicopathological features are found to be intermediate between IBC-NST and ILC, the correlation with ILC features is higher [10,13]. This is also demonstrated by the metastatic spread of mixed IBC-NST/ILC, that seems to be more similar to ILC including metastases to the peritoneum, gastro-intestinal and genitourinary tract [10].

The biological features of mixed IBC-NST/ILC have been the interest of only a few publications, which demonstrated that primary mixed IBC-NST/ILC tumors do not have distinct molecular features in comparison with ILC and IBC-NST [[14], [15], [16]]. While these studies were adding insights on primary tumors, metastatic lesions were not examined.

To our knowledge, no extensive histological comparison of primary mixed IBC-NST/ILC with subsequent metastatic lesions has been done. In the present study, we therefore aimed at evaluating the histology of all metastases from two patients with a primary mixed IBC-NST/ILC who consented to post-mortem tissue donation through our UPTIDER program (NCT04531696).

## 2 Methods

Considering only samples with a cellularity of ≥10 %, we included 14 metastatic samples originating from 7 different sites (brain, abdominal lymph nodes, axillary lymph nodes, cervical lymph nodes, pleura, retroperitoneal connective tissue and visceral fat) from UPTIDER patient #2015 and 43 metastatic samples originating from 14 different sites from UPTIDER patient #2018 (adnexa, adrenal gland, bladder, contralateral breast, intestines, abdominal lymph nodes, axillary lymph nodes, thoracic lymph nodes, peritoneum, retroperitoneal connective tissue, stomach, subcutaneous metastasis, uterus and visceral fat). Two pathologists (G.Z. and G.F.) reviewed H&E stained slides and annotated E-cadherin IHC (clone NCH-38, 1:50, Dako, CE-IVD) staining patterns as follows: preserved (complete membranous), aberrant (partially membranous, cytoplasmic, perinuclear) or absent. β-catenin IHC (clone ß-catenin-1, ready-to-use, Dako) was annotated in a similar way. Beside morphological features, the presence of IBC-NST component was confirmed by a preserved staining pattern, while an aberrant or absent stain was indicative of a lobular-like or ILC component [10].

## 3 Results and discussion

**Fig. 1:**
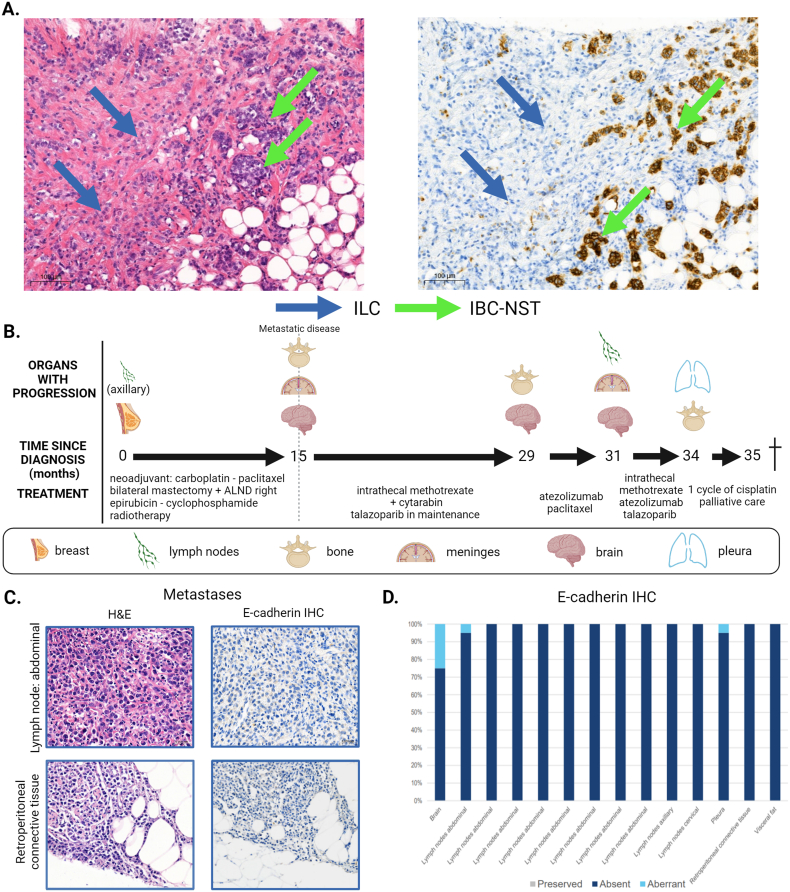
**Clinical course and histological features of primary and metastatic disease in patient #2015.** The H&E (left) and immunohistochemical E-cadherin staining (right) of the same area after multiple sections of the CNB are shown in **panel A**. The CNB showed a triple-negative, grade 3 tumor consisting of 60 % ILC (blue arrows) and 40 % IBC-NST (green arrows). **Panel B** summarizes the clinical course from the diagnosis of metastatic disease until death. The H&E and E-cadherin (NCH-38, Dako) staining of two metastatic examples are shown in **Panel C**. **Panel D** gives an overview of the number of cells with preserved, aberrant, or absent E-cadherin per metastatic lesion, no cells were found to have preserved staining. ALND: axillary lymph node dissection; CNB: core needle biopsy; H&E: hematoxylin and eosin; IBC-NST: invasive breast carcinoma of no special type; IHC: immunohistochemistry; ILC: invasive lobular carcinoma. Created with BioRender.com (For interpretation of the references to colour in this figure legend, the reader is referred to the Web version of this article.)

Since patient #2015, diagnosed with a cT4dN1M0 grade 3 triple negative BC, received neo-adjuvant chemotherapy, only the core needle biopsy (CNB) was representative for the untreated primary tumor. While it was reported as an ‘IBC-NST with lobular features’ in an external hospital, central revision of the CNB revealed that the tumor consisted of 60 % of ILC with a proliferation index of 45 % and 40 % of IBC-NST with a proliferation index of 70 % (Fig. 1A) and was thus a mixed IBC-NST/ILC. Central review of the resection specimen revealed that the residual disease in breast and axillary lymph nodes consisted only of few ILC cells (RCB-I, Miller Payne score 4), confirmed by E-cadherin IHC.

**Fig. 2:**
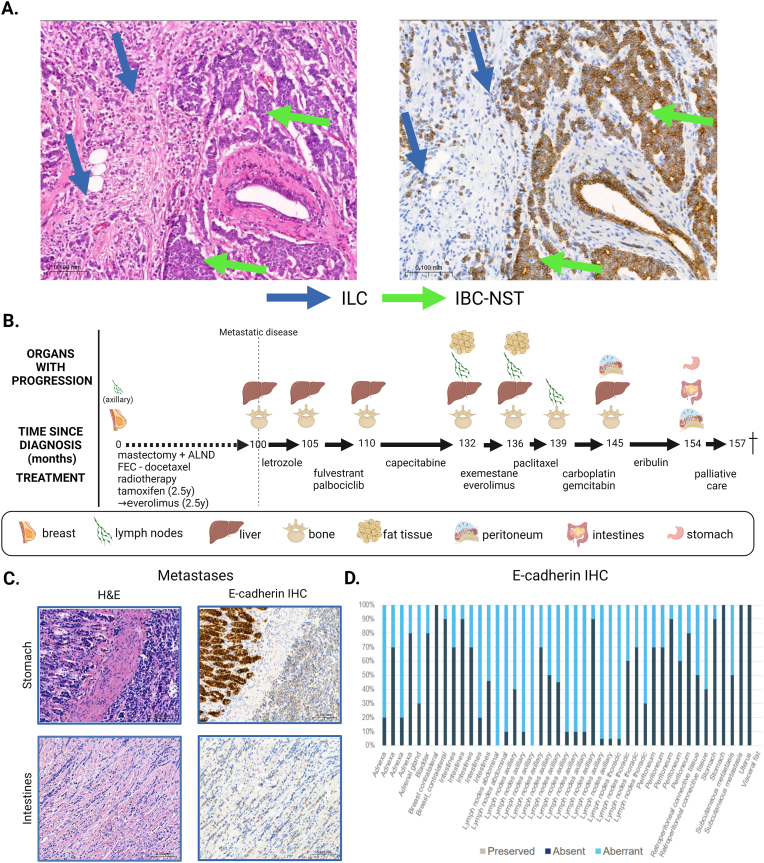
**Clinical course and histological features of primary and metastatic disease in patient #2018.** The H&E (left) and immunohistochemical E-cadherin staining (right) of the CNB are shown in **panel A** (with internal control). The CNB showed a hormone receptor positive, HER2 negative grade 2 tumor consisting of 25 % ILC (blue arrows) and 75 % IBC-NST (green arrows). **Panel B** summarizes the clinical course from the diagnosis of metastatic disease until death. The H&E and E-cadherin (NCH-38, Dako) staining of two metastatic examples are shown in **Panel C**. **Panel D** gives an overview of the number of cells with preserved, aberrant or absent E-cadherin per metastatic lesion, no cells were found to have preserved staining. ALND: axillary lymph node dissection; CNB: core needle biopsy; H&E: hematoxylin and eosin; HER2: human epidermal growth factor receptor 2; IBC-NST: invasive breast carcinoma of no special type; IHC: immunohistochemistry; ILC: invasive lobular carcinoma. Created with BioRender.com (For interpretation of the references to colour in this figure legend, the reader is referred to the Web version of this article.)

At diagnosis, the biopsy result from patient #2018, diagnosed with a non-metastatic pT2mN1a grade 2, ER+/HER2- BC, was reported as an ‘IBC-NST with lobular features’ but reclassified as mixed IBC-NST/ILC by central review. The CNB of patient #2018 consisted of 25 % ILC and 75 % IBC-NST, which was confirmed on the surgical specimen of the primary tumor. (Fig. 2A). The disease courses of both patients are summarized in Fig. 1, Fig. 2 respectively.

Literature suggests that the predominant histological type of the primary tumor, correlates with the histological type of the metastases [17]. However, in accordance to Naszaradini et al., histological examination of all the samples showed for both patients pure ILC histology and the E-cadherin staining pattern was either completely absent (9/14 for patient #2015 and 4/43 for patient #2018) or a mixture of absent and aberrant patterns (5/14 for patient #2015 and 39/43 for patient #2018) as demonstrated in Fig. 1C and D and Fig. 2C and D, both patterns being consistent with ILC diagnosis. Examples of β-catenin IHC confirming the ILC histology in the metastases of both patients can be found in Supplementary Fig. 1.

Although only two patients with mixed IBC-NST/ILC have been included in UPTIDER so far, we were able to sample an unprecedented number of metastases that display predominantly a lobular histology, independent of the predominant subtype of the primary tumor. This is consistent with previous publications which indicate that clinicopathological features of mixed IBC-NST/ILC are more closely related to ILC than to IBC-NST [10,13].

## 4 Conclusions

To conclude, our research suggests that there should be awareness of a possible predominant lobular metastatic component in patients with mixed IBC-NST/ILC. Clinicians should be aware of symptoms related to ILC metastases (e.g. bloating, gastro-intestinal problems, etc.) in patients with primary mixed IBC-NST/ILC. Therefore, our results challenge current preferred imaging modalities for metastatic detection and may suggest use of more sensitive technique such as WB-DWI MRI, requiring clinical validation in larger cohorts or patients with primary pure ILC and mixed IBC-NST/ILC. Since metastatic spread is similar to pure ILC, WB-DWI MRI or FES PET/CT could be considered for the detection of metastatic disease for patients with primary mixed IBC-NST/ILC. Additionally, these patients might benefit from ILC specific treatments, like ROS1 inhibitors along with other agents that are currently being investigated [2]. In the future, clinical trialists could also consider including patients with mixed IBC-NST/ILC in studies for patients with metastatic ILC.

## List of abbreviations

• 18F-FDG PET/CT: [18F]2-fluoro-2-deoxy-d-glucose positron emission tomography/computerized tomography
• BC: breast cancer
• CNB: core needle biopsy
• CT: computerized tomography
• ER: estrogen receptor
• FES PET/CT: fluoro-estradiol (FES) PET/CT
• H&E: hematoxylin and eosin
• IBC-NST: invasive breast carcinoma of no-special type
• ILC: invasive lobular carcinoma
• WB-DWI MRI: whole body diffusion weighted MRI

## Ethical approval and patient consent

This study was approved by the local ethics committee of UZ/KU Leuven (NCT04531696, local ethics number: S64410, approval November 30, 2020). Participating patients consented prior to death to the retrieval of archived samples, clinical data and participation in the post-mortem tissue donation program.

## Data availability

The datasets used and/or analysed during the current study are available from the corresponding author on reasonable request.

## Funding

This study was funded by the Klinische Onderzoeks-en Opleidingsraad (KOOR) of 10.13039/100012324University Hospitals Leuven (Uitzonderlijke Financiering 2020) and C1 of 10.13039/501100004040KU Leuven (C14/21/114). Additionally, K.V.B. is funded by the 10.13039/100000982Conquer Cancer – Lobular 10.13039/100001513Breast Cancer Alliance Young Investigator Award for Invasive Lobular Carcinoma Research, supported by Lobular 10.13039/100001513Breast Cancer Alliance. Any opinions, findings, and conclusions expressed in this material are those of the author(s) and do not necessarily reflect those of the American Society of Clinical Oncology® or Conquer Cancer®, or Lobular 10.13039/100001513Breast Cancer Alliance. M.D.S., K.B. and J.V.C are funded by the 10.13039/501100004040KU Leuven Fund Nadine de Beauffort; T.G., F.R. (1297322 N) and H.W. by the 10.13039/501100003130Research Foundation Flanders (10.13039/501100003130FWO); M.M. and H-L.N. by the 10.13039/501100000781European Research Council (10.13039/100016882ERC, FAT-BC 101003153).

## CRediT authorship contribution statement

**Gitte Zels:** Writing – review & editing, Writing – original draft, Visualization, Resources, Methodology, Investigation, Funding acquisition, Data curation, Conceptualization. **Karen Van Baelen:** Investigation, Funding acquisition, Data curation, Conceptualization, Methodology, Resources, Visualization, Writing – original draft, Writing – review & editing. **Maxim De Schepper:** Writing – review & editing, Resources, Methodology, Data curation, Investigation. **Kristien Borremans:** Writing – review & editing, Resources. **Tatjana Geukens:** Funding acquisition, Project administration, Resources, Writing – review & editing. **Edoardo Isnaldi:** Resources, Writing – review & editing. **Hava Izci:** Resources, Writing – review & editing. **Sophia Leduc:** Resources, Writing – review & editing. **Amena Mahdami:** Resources, Writing – review & editing. **Marion Maetens:** Funding acquisition, Project administration, Resources, Writing – review & editing. **Ha Linh Nguyen:** Writing – review & editing, Resources. **Anirudh Pabba:** Writing – review & editing, Resources. **François Richard:** Resources, Writing – review & editing. **Josephine Van Cauwenberge:** Resources, Writing – review & editing. **Ann Smeets:** Resources, Writing - review & editing. **Ines Nevelsteen:** Resources, Writing - review & editing. **Patrick Neven:** Resources, Writing – review & editing. **Hans Wildiers:** Resources, Writing – review & editing. **Wouter Van Den Bogaert:** Methodology, Writing – review & editing. **Giuseppe Floris:** Conceptualization, Funding acquisition, Investigation, Methodology, Resources, Supervision, Writing – review & editing. **Christine Desmedt:** Writing – review & editing, Writing – original draft, Resources, Project administration, Methodology, Investigation, Funding acquisition, Data curation, Conceptualization.

## Declaration of Competing interest

All authors declare to have no conflict of interest
