# Supplementary material for: Metastases of primary mixed no-special type and lobular breast cancer display an exclusive lobular histology
Source: Breast. 2024 Apr 12;75:103732. doi: 10.1016/j.breast.2024.103732 (PMC11053301; doi:10.1016/j.breast.2024.103732)
Supplement: Multimedia component 1 [file mmc1.docx]

**Appendix: Metastases of primary mixed no-special type and lobular breast cancer display an exclusive lobular histology**

Zels G., Van Baelen K. *et al.*

Overview:

- Supplementary Figure 1: growth pattern, E-cadherin IHC and Beta-catenin IHC of primary and metastatic lesions of patient 2015 and 2018

**Supplementary Figure 1: growth pattern, E-cadherin IHC and Beta-catenin IHC of primary and metastatic lesions of patient 2015 and 2018**


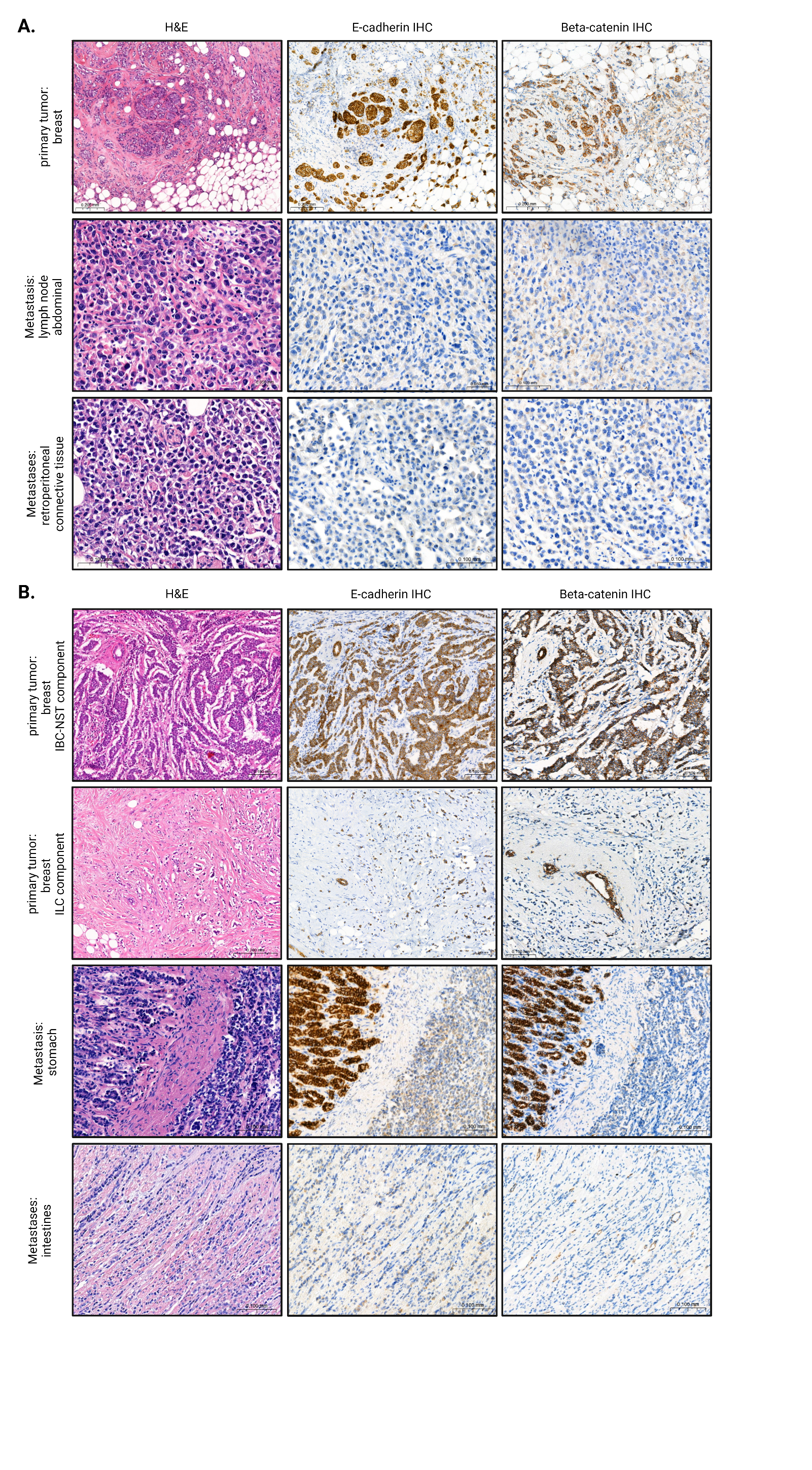


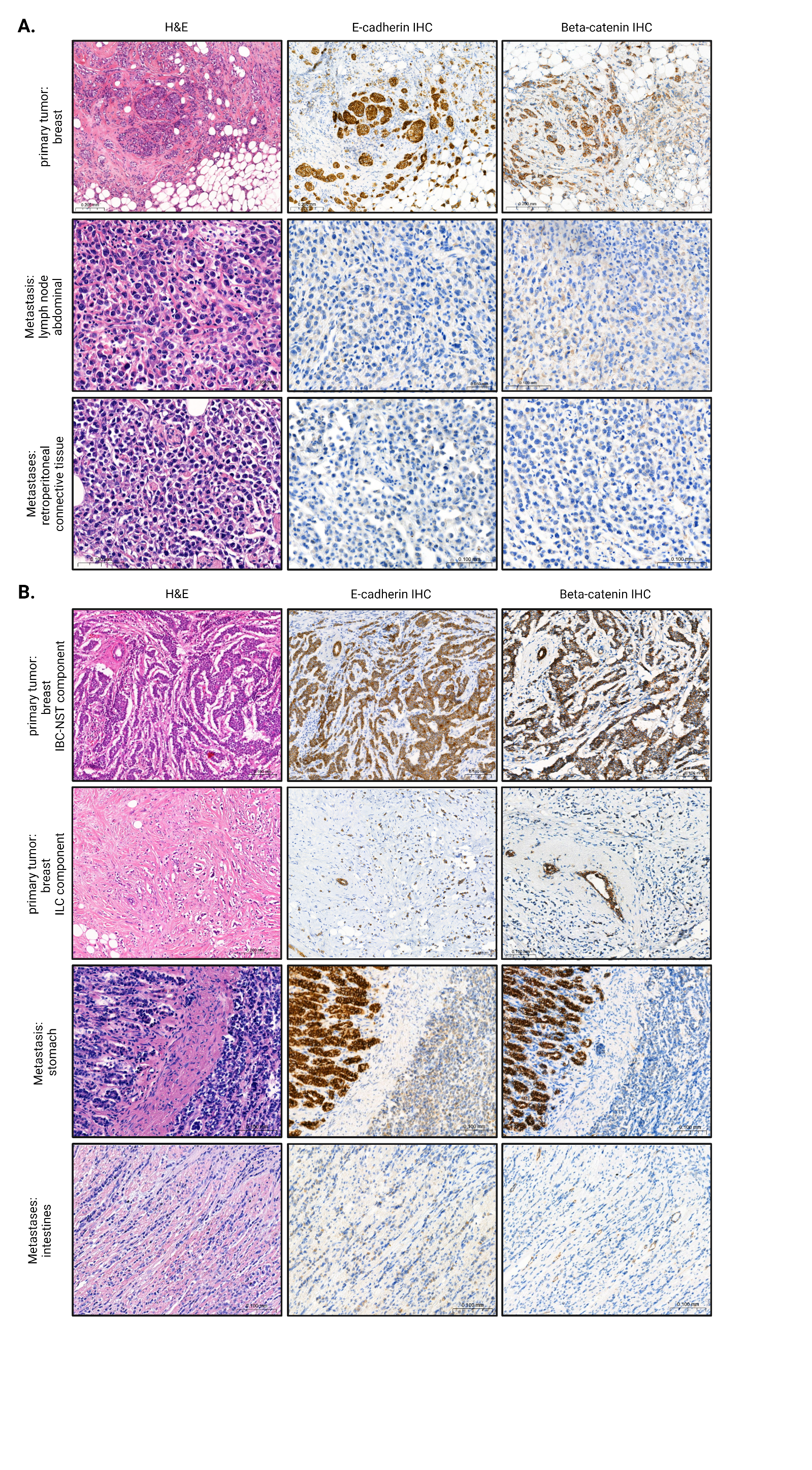


The H&E (left) and immunohistochemical E-cadherin (middle) and Beta-catenin staining (right) of the same area after multiple sections of the primary tumor (upper) and examples of metastases (middle and lower) are shown for patient 2015 in **panel** **A** and 2018 in **panel B**. NCH-38 (Dako) was used for the staining of E-cadherin and ß-catenin-1 (Dako) was used for the staining of beta-catenin.
H&E: hematoxylin and eosin; IBC-NST: invasive breast carcinoma of no special type; IHC: immunohistochemistry; ILC: invasive lobular carcinoma
